# Editorial: Understanding conscious experiences during sleep

**DOI:** 10.3389/frsle.2026.1985086

**Published:** 2026-10-12

**Authors:** Adriana Michalak, Claudia Picard-Deland, Giulio Bernardi, Mohamed S. Ameen

**Affiliations:** 1 Department of Biomedical, Metabolic, and Neural Sciences, University of Modena and Reggio-Emilia, Modena, Italy; 2 Paris Brain Institute, Hôpital Universitaire Pitié-Salpêtrière, Paris, France; 3 IMT School for Advanced Studies Lucca, Lucca, Italy; 4 Laboratory for Sleep, Cognition and Consciousness Research, Department of Psychology, University of Salzburg, Salzburg, Austria; 5 The Siesta Group Schlafanalyse GmbH, Vienna, Austria

**Keywords:** automatic dream analysis, conscious experiences, dream engineering, dreaming, lucid dreaming, natural language processing, out-of-body experience, phenomenology

The sleeping brain hosts a spectrum of subjective experiences, ranging from fleeting hypnagogic images and simple thoughts to vivid, full-fledged dreams and lucid dreaming. The works published in this Research Topic present novel methodological and conceptual approaches to studying these experiences. Contributions span the challenges of studying dreaming in clinical populations (Fischmann et al.), considerations around the boundaries of altered states of consciousness (Weiler et al.), computational dream report analysis (Bertolini et al.), and dream engineering (Haar Horowitz et al.).

## Methodological challenges

In their methodological report, Fischmann et al. recruited patients with ischemic stroke of the posterior cerebral artery (PCA) and intended to use the lesion as a model to investigate the loss of dreaming capacity. Cessation of dreaming in these patients would provide causal evidence for the role of parieto-occipital regions, moving beyond the largely correlational nature of previous work (e.g., Siclari et al., 2017). The study aimed to compare PCA patients who had lost the ability to dream with those who retained it, to investigate whether the loss of dreaming affects sleep quality and memory consolidation.

Out of 255 patients screened, 14 (5.5%) met the inclusion criteria, and only six completed the study. Importantly, the participants who completed the study were generally younger, had milder strokes, and better functional outcomes than non-completers. This recruitment pitfall alone provides a valuable insight, illustrating the clinical complexity, multimorbidity, and rarity of isolated PCA lesions that make this population difficult to study in the context of dream research. The authors suggest that future investigations could instead use approaches such as transcranial alternating current stimulation (tACS) in healthy volunteers to create *temporary functional lesions*, overcoming some of the challenges inherent to pathological models.

## Phenomenological characterization

Beyond recruitment choices, another challenge lies in *how* sleep conscious experiences are defined in the first place. Conscious experiences are inherently subjective, which requires precise language and definitions to make the findings comparable across studies. Weiler et al. examine the phenomenological boundaries between out-of-body experiences (OBEs) and lucid dreaming (LDs), asking whether these represent distinct categories or variations of the same underlying experience. The authors reviewed experiential features previously proposed to differentiate OBEs from LDs, examining both the phenomenological characteristics and interpretive frameworks used to categorize these experiences. They suggest that *disembodiment*—the subjective experience of being detached from the physical body—should serve as the primary distinguishing feature of OBEs. This work aims to provide a more standardized classification strategy, regardless of other phenomenological features of the experience, which may not be unique to or consistently reported in OBEs.

## Computational dream report analysis

Once experiences are characterized, the question becomes *how* to analyze large corpora of subjective experience reports. Natural Language Processing (NLP) and Artificial Intelligence (AI) tools are of considerable interest, as they allow reproducible and automated extraction of quantitative metrics from narrative dream reports. Manual content annotation is time-consuming, can be subject to cognitive bias and is prone to high inter-rater variability. AI-based approaches, however, can accelerate and standardize linguistic-level analysis, thereby increasing scalability. Yet, it was suggested that these tools might be poorly suited for modeling dream reports because of their unusual and bizarre content. The work by Bertolini et al. tests this claim using *perplexity*, a standard AI metric that estimates model uncertainty. By comparing the perplexity of Large Language Models (LLMs) on dream reports from DreamBank with that of Wikipedia articles, the authors show that dream content can be as predictable as Wikipedia entries. Interestingly, perplexity scores also reflected previously observed group differences in dream reports, such as gender, visual impairment and clinical status. These findings challenge the view that dream reports are inherently more bizarre or unpredictable than standard texts and offer evidence in support of AI tools for dream analysis.

## Dream engineering

While computational approaches automate analysis of dream content, dream engineering aims to manipulate and track the content of the experience *itself*, offering a systematic approach to investigate dreams despite their considerable variability. As such, in addition to standardizing the definitions and methods used to study dreams, researchers are beginning to explore ways of standardizing dreams *themselves*. In their work, Haar Horowitz et al. used a targeted dream incubation (TDI) technique involving verbal prompts and serial awakenings—at sleep onset and after entry into REM sleep—to evoke dreams about a specific topic, in this case, a tree. The experimental set-up used the Dormio system, designed specifically to facilitate TDI by detecting sleep onset based on physiological proxies and delivering the verbal prompt: “*Remember to think of a tree*.”

All 11 participants successfully incorporated the target *tree* theme into their sleep-onset dreams. Additionally, the authors provide preliminary evidence that TDI may extend beyond the hypnagogic state and influence dream experiences in subsequent REM sleep. TDI is a promising technique to investigate how dreams are generated and how they evolve across sleep stages. Perhaps most interestingly, the ability to steer dream content opens the possibility of experimentally testing hypotheses about the functions of dreams, for example, whether inducing positive dream content improves next-day mood, or whether dreaming about a desired object changes waking desire for it.

## Summary and future directions

Several key directions for future research emerge from this Research Topic. The most foundational is terminological: establishing clearer definitions and categories of conscious sleep experiences will allow studies to consistently identify and compare the phenomena they investigate.

Negative and null findings should be reported systematically. As Fischmann et al. show, recruitment challenges alone can nonetheless yield valuable methodological insights.

Finally, progress in dream engineering will require improving success rates, determining the extent and limits of control over dream content, and more directly assessing its possible applications and potential side effects.

Together, this multidisciplinary Research Topic outlines how the field is moving from *simply* analyzing dreams toward treating conscious sleep experiences as phenomena that can be systematically characterized, computationally modeled, and potentially manipulated. As these technological approaches advance in capability—particularly in the ability to actively shape dream content—ethical frameworks in consciousness research must evolve alongside them, or ideally, stay one step ahead, guarding dreams as dreams guard our sleep.
